# Cognitive dysfunction in diabetes: how to implement emerging guidelines

**DOI:** 10.1007/s00125-019-04977-9

**Published:** 2019-08-16

**Authors:** Geert J. Biessels, Rachel A. Whitmer

**Affiliations:** 1 Department of Neurology, G03.232, UMC Utrecht Brain Center, University Medical Center Utrecht, PO Box 85500, 3508 GA Utrecht, the Netherlands; 2 Department of Public Health Sciences, Division of Epidemiology, Population Brain Health Laboratory, University of California Davis, Davis, CA USA

**Keywords:** Alzheimer’s disease, Cognitive dysfunction, Dementia, Guidelines, Implementation, Mild cognitive impairment, Review, Screening, Type 1 diabetes, Type 2 diabetes

## Abstract

Cognitive dysfunction, including mild cognitive impairment and dementia, is increasingly recognised as an important comorbidity and complication of diabetes that affects an individual’s well-being and diabetes management, and is associated with diabetes treatment-related complications. Recent guidelines therefore recommend screening for cognitive impairment in older individuals with diabetes. In addition, these guidelines suggest that glucose-lowering treatment should be tailored in those diagnosed with cognitive impairment, to reduce the risk of hypoglycaemia and improve treatment adherence. This review gives an overview of cognitive dysfunction in people with diabetes, briefly describing the clinical features of different stages of cognitive dysfunction and their epidemiology. In particular, it addresses essential additional steps that need to be taken to fully implement the emerging guidelines on screening and management of cognitive dysfunction in diabetes into daily practice.

## Introduction

Cognitive dysfunction is an important comorbidity of diabetes. This may reflect brain changes as a consequence of diabetes [1] but the co-occurrence of diabetes and cognitive dysfunction clearly also reflects shared risk factors, the most obvious of which is age. Currently, the worldwide prevalence of diabetes in people older than 65 years is 18.8% and the 2017 estimate of the number of people aged 65–99 years living with diabetes around the world was 122.8 million [2]. This is expected to double over the next three decades, primarily due to the increase in the number of older people [2]. For dementia, population trends are similar. The worldwide prevalence of dementia in people older than 60 years is 6–7%, with limited variation between different regions in the world [3]. According to 2015 estimates, there were at that time 46.8 million people living with dementia around the world, with an expected doubling in the next two decades [3]. Data from a large veteran’s registry in the USA showed that, among people with diabetes, the prevalence of dementia and cognitive impairment combined was 13.1% for those aged 65–74 years and 24.2% for those aged 75 years and older [4].

Although both individuals with diabetes and their physicians are increasingly aware of cognitive dysfunction in relation to diabetes, this awareness still lags behind that of other diabetes complications [5]. Patients report that their healthcare providers sometimes have difficulty addressing cognitive dysfunction in relation to diabetes [6]. Professional diabetes societies do recognise this important knowledge gap and increasingly include information on cognitive dysfunction in educational activities. Moreover, because it is clear that particularly the more severe stages of cognitive dysfunction affect many aspects of life, including diabetes management, professional guidelines on medical care in diabetes also increasingly address cognition [7–11]. This is an important development but (as we will argue) additional steps need to be taken before these guidelines can be fully put into practice. In this review, we summarise the different manifestations of cognitive dysfunction in adults with diabetes, both in terms of clinical features and epidemiology. We also address current evidence on the impact of cognitive dysfunction on diabetes management. Finally, we discuss the emerging guidelines and address knowledge gaps and further actions that should be taken for full implementation.

## Manifestations of cognitive dysfunction in adults with diabetes

In this review, the term ‘cognitive dysfunction’ in relation to diabetes refers to any deviation of cognitive functioning compared with people without diabetes. Of note, cognitive dysfunction in people with diabetes is not a unitary construct. It is important to distinguish between ‘cognitive impairment’, which refers to dysfunction that is severe enough to be classified as ‘abnormal’ at an individual patient level based on normative cognitive test values, and more subtle forms of dysfunction wherein the mean performance of people with diabetes as a group is lower than that of people without diabetes but does not meet formal criteria for abnormal test scores.

### Dementia

Dementia is the most severe of the different stages of cognitive dysfunction, with objective impairment of multiple cognitive domains, by definition affecting activities of daily life. Most of the epidemiological work to date has focused on type 2 diabetes and dementia, with estimates in risk increase ranging from 50% to 100% [12]. A study in 2016 analysed pooled data from 14 studies comprised of over 2.3 million people [13]. This study analysed over 100,000 occurrences of dementia and found a 60% increased risk for all-cause dementia; when limiting the outcome to ‘non-vascular dementia’, mostly defined as clinically diagnosed Alzheimer’s disease, the risk increase was 50%. Interestingly, the magnitude of risk increase for diabetes on vascular dementia was 18% greater in women than in men [13] but it is unclear whether this reflects a true sex difference in the impact of diabetes on dementia risk or whether this is an artefact of selective survival and increased longevity in women.

Epidemiological data on type 1 diabetes and dementia is relatively sparse. This is because type 1 diabetes is much less common than type 2 diabetes and individuals with type 1 diabetes have only recently been living to old age [14]. Hence, late-life cognitive dysfunction and dementia risk is a more recent consideration for those with type 1 diabetes. The largest study to date in type 1 diabetes is a retrospective cohort study of individuals hospitalised for type 1 or type 2 diabetes and risk of dementia [15]. This study examined risk of dementia in over 300,000 people with type 1 diabetes, 1.8 million people with type 2 diabetes and a reference cohort. Those with type 1 diabetes had a 65% increased risk of dementia and those with type 2 diabetes had a 37% increased risk, suggesting that the risk increase is somewhat larger for those with type 1 diabetes. Thus far, no studies have delineated type 1 diabetes and risk of vascular vs non-vascular dementia. Clearly, additional studies are needed but evidence to date suggests that, on a population level, those with either type 1 or type 2 diabetes have a 40–60% increased risk of all-cause dementia.

### Mild cognitive impairment

Mild cognitive impairment (MCI) is defined as acquired cognitive complaints with objective abnormal test results in one or more domains on formal cognitive testing. The primary distinction from dementia is that, by definition, in MCI cognitive deficits should not (or only minimally) interfere with instrumental activities of daily living [16]. MCI can be further categorised into memory-impaired (amnestic) MCI vs non-memory-impaired MCI. When compared with individuals without MCI, those with MCI have an increased risk of dementia (meta-analysis: RR 3.3 [16]), although not everyone with MCI will get dementia and, in some people, cognition may even revert back to normal.

Fewer population-based studies have explored the association between type 2 diabetes and increased risk of MCI. A meta-analysis identified two studies including 393 individuals with type 2 diabetes showing a 20% pooled increased risk of MCI [17]. Research also indicates that type 2 diabetes further increases the rate of conversion from MCI to dementia, possibly accelerating the process, though there is heterogeneity in these findings for amnestic MCI vs non-amnestic MCI [18]. To date, no such studies have been carried out in individuals with type 1 diabetes. Given the preliminary data on type 1 diabetes and dementia, one can also expect that those with type 1 diabetes are also at increased risk of MCI. More research is needed on the continuum of cognitive ageing, including progression to MCI, among individuals with type 1 diabetes. Given the decreased life expectancy of individuals with type 1 vs type 2 diabetes, the earlier age of diabetes onset and the higher exposure to acute hypoglycaemia and microvascular complications, it is plausible that the continuum of MCI to dementia may vary according to type of diabetes.

### Diabetes-specific cognitive decrements

Type 1 and type 2 diabetes are both associated with subtle so-called cognitive decrements [19]. These decrements are defined as a deviation from normal cognitive functioning but, unlike MCI, this deviation is not severe enough to be classified formally as cognitive impairment [19]. In type 2 diabetes, these decrements usually manifest themselves in a cognitive test performance result on average a one-third to one-half SD lower than in those without diabetes [20]. These subtle decrements may impact all cognitive domains in type 2 diabetes, including memory, processing speed and executive function, and appear to be present at all ages [21], although clearly in most individuals type 2 diabetes has a mid- to late-life onset.

In type 1 diabetes, the subtle cognitive decrements are slightly larger and impact some domains more than others. Systematic reviews posit a one-third to three-quarter SD reduction overall, with effects most pronounced in mental flexibility, general intelligence and psychomotor speed [22]. An earlier age of onset and longer duration are risk factors for worse cognitive performance in the type 1 diabetic population [23]. While type 1 diabetes is mainly diagnosed in childhood or adolescence, it can also develop in mid and late life. It is not known whether individuals with older age of onset of type 1 diabetes exhibit the same pattern of impairment seen in the typical childhood-onset population of type 1 diabetes.

## Risk factors for cognitive dysfunction in diabetes

For both type 1 and type 2 diabetes, poor glycaemic control (including glycaemic variability), hypoglycaemic and hyperglycaemic events, age, depression and vascular complications are associated with increased risk of dementia (in type 2 diabetes) and worse cognitive performance (type 1 and type 2 diabetes) [19, 23–26]. Thus, there is heterogeneity in individual risk increase for significant cognitive impairment in the population with diabetes. Of note, there is currently no evidence that intensified glycaemic control has benefit (or harm) for preserving cognitive functioning in people with type 1 [27] or type 2 diabetes [26, 28]. Observational data suggest that some glucose-lowering agents may be associated with lower dementia risk than others, but this needs to be regarded with caution as confounding by indication may be an important issue [29].

## Impact of cognitive dysfunction in diabetes

Cognitive dysfunction, most evidently for MCI, and even more so for dementia in more advanced cognitive impairment stages, has a major impact on people’s life. This is clearly not specific to diabetes. However, some aspects of cognitive dysfunction, particularly in relation to an individual’s self-management of disease, are relevant for those with diabetes. Glucose monitoring, having to follow often-complicated medication regimens and keeping those aligned with one’s diet and exercise require planning, oversight and sometimes complex decision making. It is not surprising that people with diabetes and cognitive impairment are more likely to perform these rather demanding tasks less well [30, 31]. Cognitive impairment also predisposes people with diabetes to treatment-related complications, such as acute severe hypo- or hyperglycaemic episodes [31, 32]. Of note, occurrence of severe hypo- or hyperglycaemic episodes also predicts future development of cognitive impairment [24, 33], indicating that the relationship between cognitive impairment and these acute metabolic emergencies is bidirectional. This is one of the reasons why guidelines advise tailoring diabetes therapy to prevent further hypoglycaemic episodes in individuals over the age of 60–65 years [9, 10]. In addition, it has been established that, compared with people with diabetes and intact cognition, those with cognitive impairment are at increased risk of major cardiovascular events and death [32].

## Emerging guidelines

In recent years, several societies have put forward guidelines on the management of older individuals with diabetes and cognitive impairment (see Text box ‘Summy of recommendations on cognitive dysfunction in diabetes from recent guidelines’ [7–11, 31]). The recommendations have two main general components: (1) cognitive impairment in individuals with diabetes should be actively sought for, because unrecognised cognitive impairment is associated with adverse health outcomes, and (2) findings should lead to an individualised diabetes management regimen, compatible with the individual’s capabilities, generally with more lenient treatment targets and simplified treatment regimens to improve treatment compliance and reduce treatment-related risks.
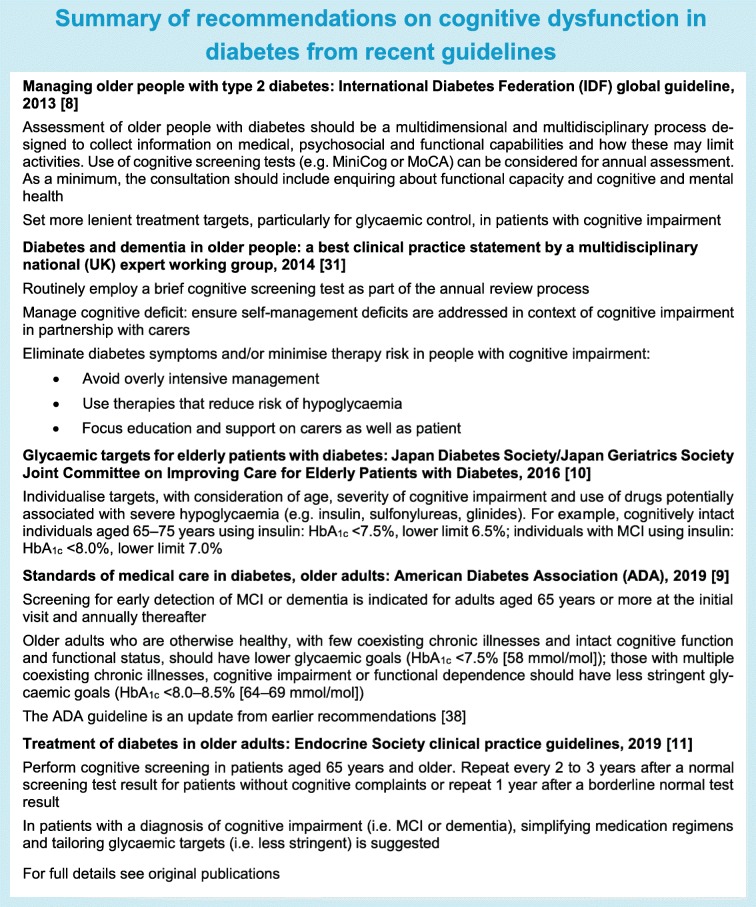


The emergence of this guidance is clearly important, as cognitive dysfunction was not really considered in diabetes management guidelines until up to a decade ago. Yet, from a practical perspective, additional steps will need to be taken to fully integrate these recommendations into daily routine.

## What is needed to bring the new guidelines into practice?

In the general population, screening for cognitive impairment is generally not recommended [34], based on the argument that no disease-modifying therapy is currently available to stop or slow down the processes that lead to dementia. Hence, early identification in people without evident complaints has even been suggested to be unethical, because an early diagnosis might be stressful while there is little to offer those that screen positive. The guidelines for people with diabetes now take a different stance, with the argument that early detection may help to avoid diabetes treatment-related risks and improve diabetes management.

Although the recommendation to screen people with diabetes for cognitive impairment seems straightforward, there are still many loose ends (summarised in the Text box ‘Suggested steps for optimal implementation of guidelines’). First, we should determine who to screen. Even brief tests generally take over 10 min to complete [35]. Implementing this for all people with diabetes is clearly labour intensive. The guidelines therefore advise primarily assessing ‘older’ people (i.e. over the age of 60–65 years) [8, 9], because the prior likelihood of detecting unrecognised cognitive impairment clearly increases with age. Yet, more individualised strategies for identifying who to screen, based on risk factor profiles beyond age, might even be more effective.
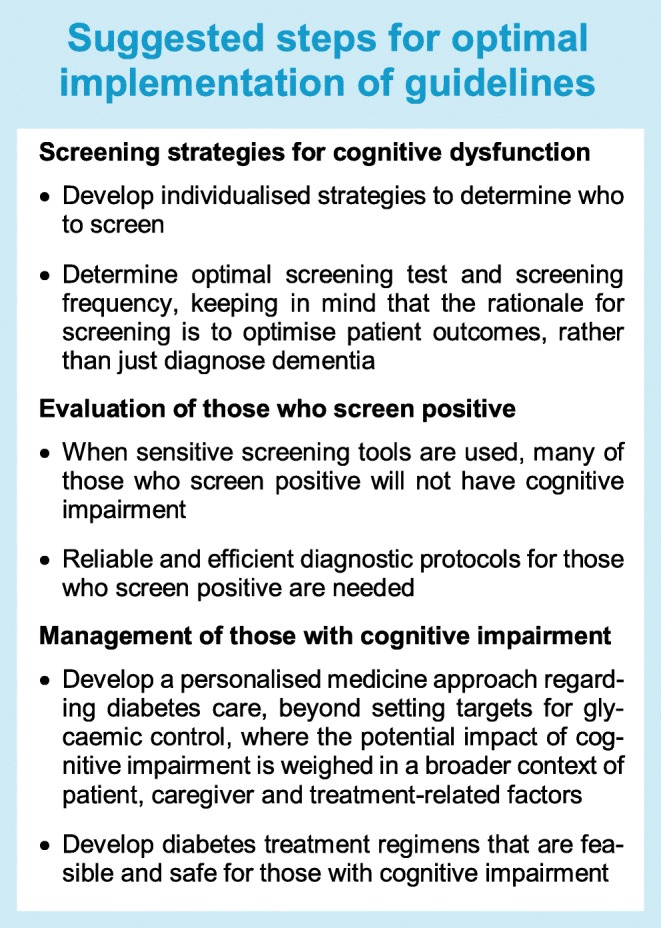


A further issue is the question of which test to use and with which cut-off. The guidelines suggest use of validated tests such as the mini-mental state examination (MMSE) or the Montreal cognitive assessment (MoCA) [8, 9]. Self-administered screening tests could be an efficient alternative [11, 36]. What is not explicitly addressed by the guidelines is whether screening should primarily try to ‘rule in’ or rather ‘rule out’ cognitive impairment. In general, most healthcare screening strategies have a multistep approach where the first step is intended to rule out the condition sought for, often by using a test with a high sensitivity, generally at the cost of specificity (i.e. lower positive predictive value). After a positive screening test, additional tests are used to establish the diagnosis (i.e. rule in). The point is that, while screening tests such as the MMSE (also depending on the cut-off used) have a reasonable sensitivity for dementia, for MCI this is much worse [35]. In a study that validated screening instruments in a population-based sample of individuals with type 2 diabetes, in which most cases identified indeed proved to have MCI, the self-administered ‘test your memory (TYM)’ and ‘self-administered gerocognitive examination (SAGE)’ questionnaires clearly outperformed the MMSE in terms of sensitivity [36]. The choice of screening tests and appropriate cut-offs for widespread and repeated use in older people with type 2 diabetes clearly warrants further evaluation. Of note, because screening aims to avoid poor outcomes of diabetes treatment in relation to cognitive impairment, sensitivity to predict these outcomes should be an essential feature of an optimal screening test.

Another key issue is how to proceed after screening. Where a screen result is negative, a repeat examination is warranted. The most recent version of the ‘Standards of medical care in diabetes’ from the ADA recommends annual assessment [9]. Yet, annual testing has the drawbacks of increased effort and costs. Moreover, most screening tests have not been developed for repeated administration and practice effects may reduce their validity. The question is therefore what screening frequency is best. Because the incidence of dementia in people with diabetes over the age of 60 years varies widely, from below 0.5% per year to over 7%, depending on age and risk factor profile [24], it might be an option to adapt the screening interval according to the patient’s individualised dementia risk.

In those who screen positive, appropriate further diagnostic evaluation is indicated. In our own study in individuals with type 2 diabetes we observed that the predictive value of a positive screening test for a diagnosis of cognitive impairment (formally established at a memory clinic) is modest, below 50% [36]. Many diabetes outpatient clinics may currently not have the expertise to make an initial distinction between true- and false-positive test results. Training will be required to avoid inappropriate referral of large numbers of patients to memory clinics.

Finally, and importantly, recommendations for the management of people with diabetes who have an established diagnosis of cognitive impairment are now predominantly based on expert opinion (see Text box ‘Summary of recommendations on cognitive dysfunction in diabetes from recent guidelines’). Although there is clear logic to these recommendations, there is evidently a need for further studies into optimal management of these individuals. This includes, but is clearly not limited to, determining optimal and safe targets for glycaemic control. With regard to the latter, the question is whether we can expect formal randomised controlled trials on glycaemic targets for people with cognitive impairment, as there may be important ethical and practical barriers to such studies. As an alternative, leveraging of large healthcare databases with longitudinal measures of glycaemic control and exposures to severe hypo- and hyperglycaemic events can be a valuable resource. Examples are to use such data to assess cumulative exposure over time to low or high HbA_1c_ and risk of diagnosed dementia [37] or include causal modelling of HbA_1c_ and vascular complications on dementia risk. Better insight into these factors can support a personalised medicine approach, where risk factors and patients’ abilities and preferences are assessed in an integral fashion to support optimal treatment.

Evidently, support and care for people with diabetes and cognitive impairment extends well beyond medical treatment. There is increasing awareness of the multifaceted impact of cognitive impairment and dementia on those affected. There are important calls for action (e.g. from the WHO) to improve care and support for people with dementia and their carers to live a life with meaning and dignity [39]. This includes efforts to make societies more dementia friendly and also, and this is clearly also relevant to the management of diabetes, to actively engage patients and their carers in policy making and the development of treatment and care approaches that are person-centred, cost-effective, sustainable and affordable and take public health principles and cultural aspects into account [39].

## Abbreviations

MCI: Mild cognitive impairment
MMSE: Mini-mental state examination
MoCA: Montreal cognitive assessment
